# Supplementary material for: Cognitive causes of ‘like me’ race and gender biases in human language production
Source: Nat Hum Behav. 2024 Jul 31;8(9):1706–15. doi: 10.1038/s41562-024-01943-3 (PMC11420078; doi:10.1038/s41562-024-01943-3)
Supplement: Supplementary file 1 — Reporting Summary [file 41562_2024_1943_MOESM1_ESM.pdf]

nature portfolio

Corresponding author(s): Jessica Brough
Last updated by author(s): Jun 4, 2024

Reporting Summary

Nature Portfolio wishes to improve the reproducibility of the work that we publish. This form provides structure for consistency and transparency in reporting. For further information on Nature Portfolio policies, see our Editorial Policies and the Editorial Policy Checklist.

Statistics

For all statistical analyses, confirm that the following items are present in the figure legend, table legend, main text, or Methods section.

n/a	Confirmed	
☐	☒	The exact sample size (*n*) for each experimental group/condition, given as a discrete number and unit of measurement
☐	☒	A statement on whether measurements were taken from distinct samples or whether the same sample was measured repeatedly
☐	☒	The statistical test(s) used AND whether they are one- or two-sided<br>*Only common tests should be described solely by name; describe more complex techniques in the Methods section.*
☐	☒	A description of all covariates tested
☐	☒	A description of any assumptions or corrections, such as tests of normality and adjustment for multiple comparisons
☐	☒	A full description of the statistical parameters including central tendency (e.g. means) or other basic estimates (e.g. regression coefficient) AND variation (e.g. standard deviation) or associated estimates of uncertainty (e.g. confidence intervals)
☐	☒	For null hypothesis testing, the test statistic (e.g. *F*, *t*, *r*) with confidence intervals, effect sizes, degrees of freedom and *P* value noted<br>*Give P values as exact values whenever suitable.*
☒	☐	For Bayesian analysis, information on the choice of priors and Markov chain Monte Carlo settings
☐	☒	For hierarchical and complex designs, identification of the appropriate level for tests and full reporting of outcomes
☐	☒	Estimates of effect sizes (e.g. Cohen's *d*, Pearson's *r*), indicating how they were calculated

Our web collection on statistics for biologists contains articles on many of the points above.

Software and code

Policy information about availability of computer code

Data collection	JSPsych library
Data analysis	R code using various libraries including dplyr and lme4

For manuscripts utilizing custom algorithms or software that are central to the research but not yet described in published literature, software must be made available to editors and reviewers. We strongly encourage code deposition in a community repository (e.g. GitHub). See the Nature Portfolio guidelines for submitting code & software for further information.

Data

Policy information about availability of data

All manuscripts must include a data availability statement. This statement should provide the following information, where applicable:
- Accession codes, unique identifiers, or web links for publicly available datasets
- A description of any restrictions on data availability
- For clinical datasets or third party data, please ensure that the statement adheres to our policy

Stimuli, data, and code for this paper can be found at https://osf.io/87vew/.

Research involving human participants, their data, or biological material

Policy information about studies with human participants or human data. See also policy information about sex, gender (identity/presentation), and sexual orientation and race, ethnicity and racism.

Reporting on sex and gender	For our studies, we recruited participants who self-reported both their sex and their gender to be either male or female. We recruited equal numbers of each group.
Reporting on race, ethnicity, or other socially relevant groupings	For our studies, we recruited participants who self-reported as Black, white, or Asian. Note that participants reported race/ethnicity both directly to us and to the Prolific marketplace; if their reports different then these participants were excluded.
Population characteristics	Participants were recruited to match requirements for sex/gender characteristics, and race/ethnicity characteristics. We did not recruit based on age except that all participants were greater than 18 years of age.
Recruitment	Participants were recruited through the online marketplace Prolific, and via social media channels (particularly WeChat and Whatsapp)
Ethics oversight	University of Edinburgh

Note that full information on the approval of the study protocol must also be provided in the manuscript.

Field-specific reporting

Please select the one below that is the best fit for your research. If you are not sure, read the appropriate sections before making your selection.

☐ Life sciences ☒ Behavioural & social sciences ☐ Ecological, evolutionary & environmental sciences

For a reference copy of the document with all sections, see nature.com/documents/nr-reporting-summary-flat.pdf

Behavioural & social sciences study design

All studies must disclose on these points even when the disclosure is negative.

Study description	Cognitive psychological experiment generating behavioural data.
Research sample	Studies 1-3 use the Prolific participant pool, and recruited English speakers. Each of these studies recruited 60 Black men, 60 Black women, 60 white men and 60 white women. The Prolific participant pool is not demographically matched to the UK or US population, but is more representative than a standard University participant pool. Study 4 recruited Chinese students at UK Universities (60 male, 60 female) who spoke both Chinese and English and were tested in one of those languages.
Sampling strategy	Participants were sampled from either Prolific or communities as above. For Study 1, the sample size was set without a power analysis, based on the maximum number of participants we could reasonably recruit with the study budget. A subsequent power analysis using the procedures described in Westfall, Kenny & Judd, (2014) and the online application jakewestfall.shinyapps.io suggests that this sample size combined with our number of trials gives 80% power to detect an effect size of 0.23. Given the robust effects found in Study 1, we continued with that same sample size in subsequent studies.
Data collection	Participants took part in online psychological experiments in which they were shown words and images.
Timing	Data was collected between 2018 and 2023, in the order described in the paper.
Data exclusions	For Experiment 1, we pre-registered to exclude trials where response times were more than 3 standard deviations from the mean, as well as trials on which participants used incorrect names or incorrect syntax, but to include trials on which participants made spelling mistakes. We pre-registered to exclude participants who took longer than 60 minutes to complete the study, who had more than 50% of their trials excluded, or who did not provide the correct syntax on at least 50% of transitive and 50% of intransitive trials. We also pre-registered to exclude participants who showed a strong orientation bias, starting their description with either the initially-left-hand-side figure on greater than 90% or less than 10% of trials. Post-hoc, we also excluded participants whose self-reported race did not match their Prolific records (n=5) and who self-reported not speaking English as a first language despite this being part of their Prolific record (n=8). The final sample comprised 196 participants (46 Black women, 45 Black men, 54 white women, 51 white men). Subsequent Experiments used the same criteria, and detailed reports of that are given in the Methods section.
Non-participation	NA
Randomization	Participants were randomly allocated to different experimental lists.

Reporting for specific materials, systems and methods

We require information from authors about some types of materials, experimental systems and methods used in many studies. Here, indicate whether each material, system or method listed is relevant to your study. If you are not sure if a list item applies to your research, read the appropriate section before selecting a response.

Materials & experimental systems

n/a	Involved in the study
☒	☐ Antibodies
☒	☐ Eukaryotic cell lines
☒	☐ Palaeontology and archaeology
☒	☐ Animals and other organisms
☒	☐ Clinical data
☒	☐ Dual use research of concern
☒	☐ Plants

Methods

n/a	Involved in the study
☒	☐ ChIP-seq
☒	☐ Flow cytometry
☒	☐ MRI-based neuroimaging
